# Calcium/calmodulin-dependent kinase II and Alzheimer’s disease

**DOI:** 10.1186/s13041-015-0166-2

**Published:** 2015-11-24

**Authors:** Anshua Ghosh, Karl Peter Giese

**Affiliations:** Maurice Wohl Clinical Neuroscience Institute, King’s College London, 5 Cutcombe Road, London, SE5 9RT UK

**Keywords:** CaMKII, Alzheimer’s disease, Post-mortem brain, Autophosphorylation, Memory, Tau

## Abstract

CaMKII is a remarkably complex protein kinase, known to have a fundamental role in synaptic plasticity and memory formation. Further, CaMKII has also been suggested to be a tau kinase. CaMKII dysregulation may therefore be a modulator of toxicity in Alzheimer’s disease, a dementia characterised by aberrant calcium signalling, synapse and neuronal loss, and impaired memory. Here, we first examine the evidence for CaMKII dysregulation in Alzheimer’s patients and draw parallels to findings in disease models which recapitulate key aspects of the disease. We then put forward the hypothesis that these changes critically contribute to neurodegeneration and memory impairment in Alzheimer’s disease.

## Background

The most common form of adult dementia, Alzheimer’s disease (AD) is characterised by progressive loss of selective cognitive functions, particularly those related to memory. It was in the early 20^th^ century that Alois Alzheimer first described the presence of ‘positive’ lesions such as senile plaques (SPs) and neurofibrillary tangles (NFTs) in the brain of a patient suffering from dementia (for a translation see [1]). However, it was not until the mid- to late 1980s that these were found to comprise aggregated amyloid-β (Aβ) peptides [2–4] and hyperphosphorylated tau protein [5–8], respectively. Although AD pathogenesis is yet to be fully elucidated, it has been posited that the Aβ peptide is central to disease onset. The “amyloid cascade” hypothesis suggests that Aβ, resulting from aberrant cleavage of the amyloid precursor protein (APP) by β- and γ-secretases, can aggregate into a toxic species, leading to a series of events that culminate in AD pathology [9, 10]. New evidence suggests alternative proteolytic pathways of APP by η-secretases can lead to production of a toxic amyloid-η (Aη) species that can also contribute to AD pathology [11].

While SPs and NFTs are useful diagnostic markers during post-mortem examination, it is actually the occurrence of ‘negative’ lesions such as synaptic loss, which precedes neuronal loss, that best correlates with the advancement of cognitive decline. Several reports have noted the progressive loss of synaptic boutons and other synaptic elements in brains of patients with symptoms ranging from mild cognitive impairment (MCI) to early-mild AD [12–17]. Hippocampal and cortical regions show the most marked loss of these features, reflecting their importance in processes of memory formation and storage. The precise mechanism by which synaptic dysfunction occurs in the AD brain is unknown; in vitro studies have shown that Aβ oligomers can directly bind to synaptic sites [18] and reduce long-term potentiation (LTP) [19–21], while facilitating long-term depression (LTD) [22]. Aβ oligomers can compromise synaptic function at both pre- and post-synaptic sites, but their early targets may in fact be excitatory post-synapses [23], where they can alter several signalling pathways.

There is significant evidence that intracellular calcium (Ca^2+^) homeostasis is disrupted in both sporadic and familial forms of AD, and can exacerbate Aβ formation and promote tau hyperphosphorylation (for reviews see [24, 25]). Additionally Aβ can influence cellular pathways involved in Ca^2+^ buffering, compromising the ability of neurons to respond to excitotoxic challenge [26], suggestive of a pathogenic feed-forward cycle where Aβ and Ca^2+^ can concomitantly impair synaptic morphology, trigger neuronal apoptosis, and eventually lead to deterioration of cognition [27]. The key players in such a pathological cascade are most likely molecules that lie downstream of Ca^2+^-signalling and are also present in excitatory synapses where Aβ oligomers likely initially bind. One candidate is the Ca^2+^/calmodulin (CaM)-dependent protein kinase II (CaMKII), the major post-synaptic protein at excitatory synapses. This kinase is fundamentally important for synaptic plasticity and memory formation. Here we discuss evidence for the involvement of CaMKII in AD pathogenesis.

## CaMKII: regulation and function

CaMKII is a holoenzyme of 12 subunits, each derived from one of four genes (α, β, γ and δ) [28]. In rat forebrain, αCaMKII and βCaMKII are the most abundant subunits, with the former expressed 3–4 times more than the latter [29], and can assemble into homo- or heteromeric holoenzymes [30]. The expression and function of α and βCaMKII differ; while α is expressed exclusively in glutamatergic neurons [31], the β subunit is also expressed in inhibitory interneurons [32]. Further, βCaMKII, but not α, binds to F-actin, which is relieved upon activation by Ca^2+^/CaM [33]. This dissociation is thought to regulate morphological changes at the synapse [34]. Functionally, αCaMKII activity is essential for synaptic plasticity and memory formation, as elegantly demonstrated in knock-in mutant mice [35]. It may also have a structural role as it can bind to various proteins at the synapse [36] and its expression is extremely abundant (about 1.4% of hippocampal protein) [29]. In contrast, βCaMKII activity is not required for synaptic plasticity and memory formation [37], indicating that the primary function of this subunit is structural.

CaMKII holoenzymes are activated by the binding of Ca^2+^/CaM, and also by NMDA receptors (NMDARs) and L-type voltage-gated Ca^2+^ channels (VGCCs) at the synapse [38]. An important aspect of αCaMKII activity is its autophosphorylation at threonine-286 (T286) (for review see [39]). This autophosphorylation results from an interaction between subunits within the holoenzyme and switches the subunit activity from a Ca^2+^/CaM-dependent to – independent state. This ‘autonomous’ activity persists at the synapse for about one minute after stimulation [38]. However, T286 autophosphorylation can last longer and the dissociation between prolonged autophosphorylation and autonomous activity is not understood [39, 40]. Studies with T286 autophosphorylation-deficient knock-in mutants have shown that this event is fundamentally important for NMDAR-dependent LTP at hippocampal CA1 synapses [41–43] but not at perforant path-granule cell synapses [44]. Furthermore, T286 autophosphorylation is essential for spatial memory formation [41, 45]. Besides T286 autophosphorylation, αCaMKII is also regulated by other autophosphorylation events, phosphatase activity and endogenous inhibitor proteins (for reviews see [36, 46]).

## CaMKII abnormalities in AD

Expression analyses of post-mortem disease brain can be very informative, in that prominent disease-related dysfunction is detectable. In contrast, studies with AD models, in rodents or in vitro, suffer from inadequate modelling of disease cause. The limitation of post-mortem studies is that they may be confounded by post-mortem delay, which can range from several hours to one day, during which protein expression may decrease and, in particular, post-translational protein modifications such as phosphorylation may be compromised. Another limitation is that they offer only one time point for analysis; however the severity of disease at the time of death can be estimated [47].

Semi-quantitative western blot studies with post-mortem tissue have suggested that αCaMKII protein expression level is not altered in hippocampus, frontal cortex or other cortical areas in the severe stages of AD [48, 49]. However, immunohistochemical analyses have indicated that αCaMKII-expressing neurons, which are excitatory, are selectively lost in hippocampal area CA1 in severe AD [50, 51] (but see [52, 53]). The remaining excitatory neurons in CA1 appear to express increased levels of αCaMKII [50, 51]. Interestingly, increased αCaMKII expression is not found in hippocampal area CA3 in severe AD [51], a region which has almost no neuronal loss in the end stages of AD, in stark contrast to substantial neuronal loss in CA1 [54].

Changes in distribution of CaMKII mRNA in AD brain are more difficult to determine. One study finds reduced hybridisation of αCaMKII mRNA in CA1, but only when neuronal loss associated with severe NFT formation is observed [55], echoing the findings of Simonian et al. However another study finds an increase in hybridisation throughout the AD hippocampus, especially in the dentate gyrus (DG) and CA3 regions [56]. A more recent microarray analysis of several brain regions from AD patients discloses that alterations in the expression of CaMKII mRNA may be far more composite than previously thought, with genes encoding different subunits showing different directions in expression changes across brain regions [57].

Early western blot studies suggest that autophosphorylation of αCaMKII at T286 is reduced in hippocampus and frontal cortex of the severe AD brain [48]. This is also reflected by the fact that cortical regions show a total loss of immunoreactivity for active conformations of CaM and reduced immunoreactivity for other forms [58]. However, this result has not been replicated [59]. Instead, it has emerged that in CA3 and the DG of AD brain, the subcellular localisation of αCaMKII autophosphorylation is altered [59]. p(T286)-αCaMKII is specifically decreased in dendrites and synapses, and increased in perikarya of CA3 neurons and granule cells of the DG. This altered distribution correlates with cognitive impairment both in patients with AD and its prodrome MCI [59]. Studies using cultured fibroblasts and lymphocytes from patients also suggest dysregulated CaMKII activity in AD [60, 61].

## CaMKII dysregulation in AD models

The study of molecular dysfunction in AD has been greatly advanced by the development of transgenic mouse models that recapitulate some AD hallmarks. However, such models usually overexpress mutated forms of the human APP gene, and therefore are not fully representative of the causes underlying AD [62]. Additionally, they are confounded by artefacts due to increased transgene expression, and ageing, the main risk factor of AD, is not sufficiently addressed. Nonetheless, if a molecular dysregulation is similar in post-mortem AD brain and in AD models, it is very likely that it occurs in the disease.

Studies on AD models, like post-mortem analyses, suggest abnormalities in regulation of CaMKII. One of the most widely used AD models is the Tg2576 mouse, which carries the APP_Swe_ mutation (K670N/M671L). While the total levels of α/βCaMKII are not altered in the frontal cortex of these mice, there is a significant alteration in their subcellular distribution, from synapse to cytosol. This change is not due to synaptic loss and is also seen in levels of active αCaMKII, suggesting a selective loss of synaptic CaMKII [63]. Another commonly used mouse model contains mutations in both APP and presenilin-1 (PS1), a component of the γ-secretase complex. Two studies find altered hippocampal expression of p(T286)-αCaMKII in these mice, and one additionally finds reduced levels of the CaMKII-binding VGCC Ca_v_1.2 and elevated CaM [64, 65]. Altered αCaMKII distribution is also found in a mouse model of sporadic AD in which amyloid oligomers are injected into the ventricles. This acute treatment results in a shift of p(T286)-αCaMKII from apical dendrites/spines to the somata of CA3 pyramidal neurons and is blocked by inhibition of the phosphatase calcineurin, which augments phosphatase-1 activity [59].

A calcineurin-dependent redistribution of autophosphorylated αCaMKII also occurs in Aβ oligomer-treated primary neuronal cultures [59, 63]. Moreover, treating hippocampal neurons with Aβ oligomers impairs αCaMKII activation [64, 66]. In contrast to rodent models, there is no change in CaM levels and greater expression of Ca_v_1.2 channels [64], a finding which is confirmed by an independent study [67]. This may be the result of cell cultures modelling earlier stages of the disease where there is no neuronal loss [64], or due to a lack of fully functional synapses.

## Impact of dysregulated CaMKII in AD

Post-mortem analyses and studies with AD models indicate that T286-autophosphorylation of αCaMKII is impaired at synapses in the disease. Considering this autophosphorylation is essential for NMDAR-dependent LTP at CA1 synapses and spatial memory formation [41, 42, 45, 68, 69], the redistribution of p(T286)-αCaMKII could contribute to cognitive impairment in AD. Consistent with this, the reduction of T286-autophosphorylation in apical dendrites of granule cells of the DG in subjects with MCI and AD correlates with cognitive dysfunction as measured by MMSE scores [59]. Moreover, spatial training of Tg2576 mice increases T286-autophosphorylation of αCaMKII in the hippocampus and rescues deficits in contextual memory formation [70], suggesting deficits in T286 autophosphorylation are key to causing impairments in synaptic plasticity and memory formation in AD. This idea is confirmed in studies with Aβ-treated cultured primary neurons, which have reduced surface expression of AMPA receptor (AMPAR) subunit GluA1 and impaired AMPAR-mediated synaptic transmission. Knockdown of CaMKII mimics these effects and CaMKII overexpression rescues these [63]. An analogous observation is seen when treating rat hippocampal slices with Aβ_1-42_, where Aβ inhibits CaMKII activation and blocks the stimulation-dependent phosphorylation of a CaMKII-specific site on GluA1 [71]. Furthermore, it has been suggested that neurotrophin-induced enhancement of p(T286)-αCaMKII leads to rescue of Aβ-induced deficits in LTP at hippocampal synapses [72].

At the neuropathological level, the finding that APP can be phosphorylated in vitro by several kinases including CaMKII [73], puts forward the hypothesis that there could be a possible link between CaMKII and Aβ production. Both McKee and Wang remark on some co-localisation of αCaMKII with SPs, with differences in the deposition pattern around diffuse and neuritic plaques [50, 51]. It has been found that phosphorylation on T668 of APP is elevated in AD brain and can regulate its cleavage by β-secretases [74], but this is not known to be a CaMKII site of phosphorylation. It has also been suggested that phosphorylation of CaMKII sites (T654/S655) can alter the conformation of APP [75] and regulate its trafficking [76], but direct evidence that CaMKII is involved is lacking.

The correlation between CaMKII and tau phosphorylation is much stronger. Increased αCaMKII expression in CA1 neurons [50, 51] and increased αCaMKII autophosphorylation in cell bodies of CA3 neurons and granule cells in the DG [59, 63] suggest that outside of synapses, αCaMKII is hyperactive. Being a tau kinase, this hyperactivity could contribute to NFT formation. NFTs are made of paired helical filaments (PHFs) which contain tau protein hyperphosphorylated at many sites [77]. Several analyses of AD brain find that αCaMKII expression in cell bodies frequently co-localises with NFTs or tau mRNA [50, 51, 53, 55, 78, 79]. Mass spectrometry has also revealed that AD brain tau is phosphorylated by CaMKII at several different sites [80]. CaMKII phosphorylation of tau alters its electrophoretic mobility and structure, in a manner specific to PHF-tau [81–83]. Additionally, isolation of PHFs from AD brains results in co-purification with αCaMKII, 4–7 times more than is observed in controls [78]. The difficulty in analysing the importance of CaMKII in tau hyperphosphorylation arises from the fact that tau can be phosphorylated by several other kinases at CaMKII sites. It has been found that phosphorylation by CaMKII alone only partially inhibits binding of tau to microtubules [84]. Additionally several post-mortem studies note that not all αCaMKII-expressing neurons develop NFTs [51, 53, 55], suggesting that other tau kinases/phosphatases are involved. A likely scenario is one where αCaMKII phosphorylation of tau can prime its phosphorylation by other kinases such as cdk5 and GSK3-β [85, 86]. Collectively, it is conceivable that CaMKII can contribute to NFT formation in AD.

The loss of synaptic proteins in AD, combined with dysregulated CaMKII, may also lead to neuronal death. It has been suggested that αCaMKII and the post-synaptic protein PSD-95 can compete for binding to the C-terminus of the NMDAR subunit NR2A upon physiological stimulus [87]. Treating hippocampal neurons with antisense oligonucleotides to PSD-95 leads to increased association of both total and p(T286)-αCaMKII with NR2A/B subunits, although the total levels of αCaMKII are unaltered [88]. This is paralleled by an increase in cell death which can be rescued by pharmacological inhibition of CaMKII. Interestingly, hippocampal neurons are more susceptible to this type of injury than cortical neurons, and in organotypic hippocampal slices, CA1 neurons show greater susceptibility than CA3 or DG neurons. This reflects the hierarchical decline of brain areas during disease progression [54], further suggesting that αCaMKII/NR2A co-expression may be a causal factor for cell death in AD. Additionally, selectively inhibiting CaMKII in Aβ-treated primary cortical cultures reduces amyloid-induced activity of caspases-2 and -3 as well as tau phosphorylation [89]. It is conceivable that the upregulation of αCaMKII in CA1 may be directly responsible for the severe atrophy seen in this region. CaMKII may also be involved in other signalling cascades related to neuronal decline [90–93].

## Conclusions

**Fig. 1:**
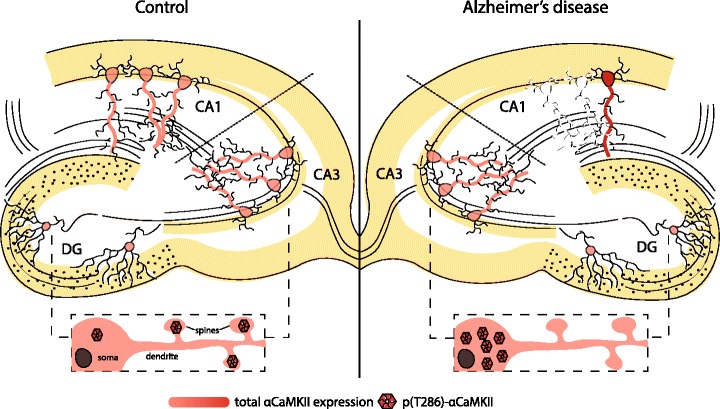
αCaMKII is dysregulated in the Alzheimer’s disease hippocampus. αCaMKII-expressing neurons are selectively lost in the hippocampal CA1 subfield in AD [50, 51], a region that shows devastating atrophy when compared to age-matched controls [54]. Remaining pyramidal neurons of this region show increased expression of αCaMKII. This increased expression may critically contribute to tau hyperphosphorylation and other neurodegenerative processes, such as caspase-3 overactivation, in CA1 pyramidal neurons [for references, see main text]. On the other hand, CA3 pyramidal neurons and granule cells of the DG do not develop these changes in total αCaMKII. They do however show a change in subcellular distribution of T286-autophosphorylated αCaMKII (inset) [59]. This change is suggested to shift CaMKII activity from the synapse to soma leading to synaptic deficits, neurodegenerative processes, and impaired memory formation. AD, Alzheimer’s disease; CA1/3, Cornu Amonis areas 1/3; αCaMKII, α subunit of calcium/calmodulin-dependent protein kinase II; DG, dentate gyrus

It has been established that CaMKII is dysregulated in AD hippocampus (Fig. 1). We suggest that this dysregulation is a key contributor to synaptic degeneration, NFT formation and memory deficits. However, the nature of CaMKII dysregulation is undoubtedly complex and several questions remain unanswered. One key question is ‘how’ this dysregulation can occur. So far, the focus has been on levels of total or T286 autophosphorylated αCaMKII. Other aspects of CaMKII regulation and activity need to be addressed, such as distribution of unphosphorylated CaMKII in AD brain, and other important sites of autophosphorylation such as T305/6. Other subunits such as β and γ may also be integral to CaMKII dysregulation. For example, βCaMKII autophosphorylation can regulate its dissociation from F-actin, thereby allowing cytoskeletal remodelling in glutamatergic excitatory synapses, a necessary occurrence for LTP induction [94]. Impaired Ca^2+^ signalling could therefore impact upon both this dissociation and the reassociation between βCaMKII and F-actin, an event crucial for stabilisation of newly remodelled actin and LTP maintenance. Additionally, γCaMKII can act as a Ca^2+^/CaM shuttle to the nucleus to alter gene expression (for review see [95]). Another fundamental issue is how CaMKII is dysregulated specifically in the CA1 region, an area showing devastating neuronal loss in AD compared to normal ageing. Is increased αCaMKII expression in remaining CA1 neurons a compensatory effect or a precursor to neurotoxicity? How does this relate to the subcellular distribution of total and p(T286) αCaMKII in CA1, and are these changes also calcineurin-dependent? Finally, it remains to be determined whether CaMKII is essential for synaptic dysfunction, cognitive impairment and NFT formation in AD. Can restoring synaptic activity of CaMKII in models of AD prevent cognitive dysfunction? Can reducing somatic CaMKII in an in vivo model of tau pathology prevent or abolish tangle formation? Elucidating these questions will investigate the hypothesis that dysregulated CaMKII is a key contributor to synaptic dysfunction, neurodegeneration and memory impairment in AD, and may point to novel treatment routes.

## Abbreviations

Aβ: Amyloid-β
AD: Alzheimer’s disease
Aη: Amyloid-η
AMPAR: α-amino-3-hydroxyl-5-methyl-4-isoxazole-propionate receptor
APP: Amyloid precursor protein
CA1/3: Cornu Ammonis areas 1/3
CaMKII: Calcium/calmodulin binding protein kinase II
cdk5: cyclin-dependent kinase 5
DG: Dentate gyrus
GluA1: AMPAR subunit
GSK3-β: Glycogen synthase kinase 3-β
LTD/LTP: Long-term depression/potentiation
MCI: Mild cognitive impairment
MMSE: Mini-mental state examination
NFT: Neurofibrillary tangle
NMDAR: N-methyl-D-aspartic acid receptor
NR2A/B: NMDAR subunits
PHF: Paired helical filament
PS1: Presenilin-1
PSD-95: Post-synaptic density protein 95
SP: Senile plaque
VGCC: Voltage-gated calcium channel
